# Economic burden of private tutoring at higher secondary level in Pakistan: An analysis through Hurder and Linear regression model

**DOI:** 10.3389/fpsyg.2022.904956

**Published:** 2022-09-28

**Authors:** Khalida Parveen, Abdulelah A. Alghamdi, Iqbal Javed, Imad U Din

**Affiliations:** ^1^Faculty of Education, Southwest University, Chongqing, China; ^2^Faculty of Education, Umm Al-Qura University, Mecca, Saudi Arabia; ^3^Department of Economics, University of Lahore, Sargodha, Pakistan

**Keywords:** economic burden, private supplementary tutoring, influencing determinants, demand, Hurder model, linear regression model

## Abstract

Economic burden of private supplementary tutoring is increasing around the world. Demand for private supplementary tutoring, contributing factors, and their effects are investigated in the study. For this purpose, data were collected through questionnaire, and multistage non-probability technique was used. Hurder model was used to find the factors affecting demand for private supplementary tutoring at the higher secondary level. Linear regression model was used to find the economic burden of family and factors affecting the demand for private tutoring. The findings showed that several factors have a significant effect on the demand for private supplementary tutoring, and some of them significantly impact on the economic burden. The study recommends regulating the market of shadow education because of differentiated tuition fee in different tuition centers to cut down the burden of private tutoring for households.

## Introduction and background

Shadow education is commonly known as private tutoring, home tuitions, or academies in Pakistan. The shadow image is found in several writings (Stevenson and Baker, 1992; Bray, 1999, 2013; Heyneman, 2011; Zheng et al., 2020; Hawrot, 2022). Specifically, shadow education refers to the supplementary and fee-paying academic teaching of students who are otherwise enrolled in institutional school/college instruction programs. Families employ private tutors to help their children with the school work generated from their formal education (Ireson, 2004; Bray, 2013; Kim et al., 2022). In 2011, Asian Development Bank (ADP) and the Comparative Education Research Centre (CERC) conducted a report that there is almost $3.40 spent on private tutoring per month on a child in Pakistan. The private tutoring business for secondary schools in Hong Kong reached $255 million, in 2011, while Japan spent $12 billion on private tutoring in 2010. Shadow education is certainly high taking place at the worldwide level because according to the Annual Status of Education Report [ASER] (2016) that in 2016–2017 tuition in private schools is 33 percent compared to 15 percent in government schools.

Across the globe, private tutoring is widely trended in various countries (Bray, 2020, 2021). The literature shows the importance of private supplementary tutoring and examines the role of private supplementary tutoring in different countries. Private supplementary tutoring has various effects which exist in the world such as mainly economic burden. According to National Cancer Institute (NCI), economic burden refers to the cost required to deal with a specific situation. It is also called economic hardship, financial burden, financial hardship, financial distress, financial stress, and financial toxicity.

Private tutoring or shadow education creates new educational opportunities but also creates heavy burden which we say it “economic burden,” on the state education system and family (Silova and Bray, 2006; Glotova et al., 2022). Participation in a fee-based activity that is adopted to enhance students’ performance could be reason for economic burden for the students and their families as well (Hajar, 2018). The basic concept of our study is the demand for private supplementary tutoring at a higher secondary level and the economic burden in Punjab province of Pakistan, especially in two districts, i.e., Lahore and Faisalabad. Different researchers have given different definitions of the term supplementary tutoring in different ways. Stevenson and Baker (1992) were the first to use the term shadow education in the title of a research article. They started their paper by giving a general definition of shadow education, saying, “Shadow education is a set of educational activities that occur outside formal schooling and are designed to enhance the student’s formal school career.” Bray (2011) defined shadow education as private supplementary tutoring of subjects that are part of core mainstream curricula (such as mathematics, language, or science). Shadow education is another name for private supplemental or additional tutoring which is suggested outside the prevailing education system. Private supplementary is broadly known as shadow education which student takes after or before school/college/university time in groups or an institution such as academies.

The system of education in Pakistan is mostly divided into six parts or levels: preschool (for the age from 3 to 5 years), primary (grades 1–5), middle (grades 6–8), secondary school level (grades nine and ten, or SSC), higher secondary school level (grades 11 and 12), university program leading to undergraduate (grades 13–14), and graduate level (grades 15–16 degrees). According to ASER 2011, the persons living in urban areas of Pakistan are usually found involved with the private tutoring. Most urban households in Pakistan are usually found involved/engaged in the private tutoring. For example, 62% of secondary students in Lahore city/district are receiving private tuition, putting it/this ratio on top of the list. Secondly, Faisalabad is at second number with 54% ratio and Peshawar is on third position in Pakistan with 34% ratio. The culture of private tuition is increasing day by day in Pakistan. Most of the students involved in private tutoring are secondary and higher secondary school students. These students are taking tuitions in different styles and ways; like, in traditional ways, it may be in one-to-one students, in groups, or at home for few hours in an informal environment. On the other hand, it may have a modern nature and convert into industry of private tuition. This private tuition industry includes large-scale business in which small- and large-scale professional centers, academies, institutions provide tuition to the students in formal ways. The increasing trend of the private tuition industry is due to its demand in the society. This demand is mainly from students and teachers. Private tuition trend or fashion has also affected the quality, utility, and functioning of the current education system. People feel dissatisfaction regarding the performance of government educational institutions. In this paper, we see perceptions of private tuition centers on quality of knowledge, teacher performance, student performance, etc., in the government education sector.

The basic concept of our study is based on the demand for private supplementary tutoring at the higher secondary level. The problem is that it is increasing day by day, and it is formulating the demand of private supplementary tutoring. With the increase of number of colleges, the number of students is also increasing; at the same time, the number of students getting private tutoring is also increasing as private tutoring is going to be a trend. Particularly in government institution, the students who do not have good academic performance are forced by their teachers and parents to get supplementary education. Though some research discloses that the demand for private supplementary tutoring entails negative impact, some argue that its positive impact is also widely acknowledged while ignoring the end result. Regarding the present study, the economic factors are also investigated to measure their effect on the demand for private tutoring in Lahore and Faisalabad districts.

Some selective subjects of interest taken by most higher secondary level students for private tutoring include mathematics, physics, English, and chemistry. It is also included in our aims to show the importance of these subjects for private supplementary tutoring. Private tuition has a positive impact on academic performance, specifically for public school students (Khan and Shaikh, 2013; Zheng et al., 2020; Kim et al., 2022). For facing high competition, they take private supplementary tutoring for taking high marks, learn study habits, and become better students. It is merely for profit creating (Zhang, 2011; Aslam and Atherton, 2012). Likewise, it is pointed out that students who received private tutoring attained an increase of 4.961 points in their standardized test scores of Chinese, math, and English (Zheng et al., 2020; Hawrot, 2022; Zhang and Liu, 2022). It has a potentially treacherous warning to impartiality (Bray, 1999; Bray and Silova, 2006). The increase in access to education has implications for inequality (Bray, 1999; Tansel and Bircan, 2004). It also increases inequalities in classrooms when some students receive tutoring but others do not.

Existing literature shows and explains different variables except the family system. As the family system is not well documented in previous studies, therefore this factor has been set as the focus of the present study. It is acknowledged through different studies that private tuition has a positive impact on academic performance, specifically for public school students (Khan and Shaikh, 2013; Fakih et al., 2022; Kim et al., 2022). For facing high competition, they take private supplementary tutoring for taking high marks. The increase in access to education has implications for inequality (Bray, 1999; Tansel and Bircan, 2004).

### Research questions

The study involves the following two research questions:-

(i) What are the factors affecting the demand for private supplementary tutoring?

(ii) What is the economic share of family income being consumed on private tuition?

## Research methodology

Dang and Rogers (2008) described that the demand and supply framework of private tutoring included the assumptions which might not always yield. Following two assumptions, this framework has: (1) in short run after a certain point public education reached to its capacity limit; (2) the market for private tutoring is competitive, and households have control over to consume or not the services of private tutoring. Regardless of these limitations, this framework presents that with the availability of private tutoring how the quantity of education increases in the market of education.

### Empirical models

In order to attain the objectives of the study, two models, Hurder model and linear regression model, were used to conduct the empirical analysis.

#### Hurder model and linear regression model

Descriptive statistics analysis and Hurder model were used to answer the given first research question. The answer to the second research question was given by the linear regression model. In this model, level of probability appears at which students were taking part in private tutoring. Students who are not participating in shadow education are spending zero expenditure on private tutoring. If students are involved in private tutoring, it means their expenditures are nor zero and this is an economic burden for their parents. In this way, the ordinary least squares (OLS) method is not suitable for the whole sample. Hurder model is considered to be more suitable if it presumes that psychological determinants play a vital role in the minds of parents while taking a decision for providing their children with private tutoring. Among these all factors, the economic factor is the most important factor in deciding the number of expenditures because long-time private tutoring caused to economic burden for parents (Silova and Bray, 2006). Likewise, the linear regression model is used to check the effect of independent variables on the dependent variables; therefore, in the present study linear regression model is used to check the effect of factors on the increasing demand for private tutoring.

In East Asia, private tutoring is considered by parents as a fundamental requirement of their children even by parents with low-income children (Lin and Chen, 2006; Lee et al., 2012; Bray and Kwo, 2013). Besides economic considerations, there are some other aspects related to private tutoring that may empower the decision of the parents for their children on taking shadow education or not, while the cost of private tutoring for a specific determine by the expenditure (Aslam and Atherton, 2012; Li, 2016) utilized Hurder to demonstrate that the expenses spent on the private tutoring hardly effect the decision of parents to provide shadow education to their children.

First step: Hurder model used for household decision to participate


(1) Li=Pi1-Pi=B+0B1X+1BX22+……….+BX10+10ei

where L_*i*_ is the Hurder model, βs is the vector of binary regression coefficients, and εi ≅ N (0, σ 2) is the error term.

Second step: Linear regression model was used to make the decision that how much expenditures were incurred regarding economic burden. It is for economic burden.


(2) $$\begin{array}{c} Y_{i}=B_{O}+B_{1}\,H\,H\,S_{i}+B_{2}\,I\,N\,C_{i}+B_{3}\,A\,G\,E_{i}+B_{4}\,G\,N\,D_{i}\\ +B_{5}\,F\,T\,G_{i}\&M\,T\,H\,E\,D\,U_{i}+B_{6}\,C\,L\,G\,T_{i}+B_{7}\,F\,M\,S_{i}\\ +B_{8}\,W\,K\,M_{i}+B_{9}\,C\,L\,G\,G\,S_{i}+B_{10}\,T\,U\,N_{i}\\ +B_{11}EXP_{i}++B_{12}FOCCP+B_{13}UC_{i},MC_{i}+e_{i} \end{array}$$

where

• L_*i*_ = Ist model dependent variable (shadow education)

• Y_*i*_ = 2nd model dependent variable (expenditure/income = economic burden)

• UC/MC = union council (rural = 0)/municipal committee (urban = 1)

• HHS = household size

• FINC = family income

• AGE = age of college/school children

• GND = gender female = 0 and male = 1

• FATH EDU = father education

• MATH EDU = mother education

• ClGT = college type, public college = 0 and private college = 1

• FMS = family system, separate family = 0 and joint family = 1

• WRKM = working member of a family

• CLGG = college-going children

• TUN = tuition, not take tuition = 0 and take tuition = 1

• EXPEND = expenditure of students who are taking tuition

• FOCCU = father occupation

•ε*i* = error term

### Research design

In this study, quantitative approach was used to collect data regarding the economic burden private supplementary tutoring at higher secondary schools in the Lahore and Faisalabad districts of Pakistan. Along with this, a face-to-face interview was organized by the authors. The factor of the illiteracy on the part of parents, especially in rural areas of Lahore and Faisalabad districts, paved the way for using this approach. That is why, a face-to-face interview through close-ended questionnaire is the best way to collect the data. The interviewees were asked to give information related to the socioeconomic status, their education level, and the related expenditures on education and expenditures regarding shadow education of their children. All the ethical codes are persuaded in the study. Parents were informed about the purpose and objectives of the research to retain confidence at all levels. The respondents were informed about their benefits and rights, and usage of data and the person is accountable for the secrecy of information.

Moreover, the obstacles in communications are extracted through translating the questionnaire in the Urdu language. The data were collected from 20 respondents for the purpose of taking pretesting under the guidance of leading supervisor. On these 20 samples, a pilot study was done with parents, and some errors and ambiguities were found. It was pointed out that tuition expenditures are not only about tuition fee but it also includes certain expenditures like stationary and transport expenditures. After removing this error, questionnaire was ready to collect the final data.

#### Data collection and sampling

Two techniques are used for data collection, namely, probability and non-probability sampling techniques. The probability sampling technique is used when the total population is known. But when the population n is not known, the non-probability sampling technique is considered more suitable. For data collection, multistage random sample technique (non-probability sampling technique) was used as total population (number of students) taking private tuitions is not known. Additionally, the Government of Pakistan did not carry a national survey in this context. Hence, the non-probability sampling technique is the best technique for the study in hand. Through structured questionnaire, data are collected from the households of Lahore and Faisalabad districts whose children are at higher secondary level college/school-going. Only the first college/school-going child is taken from that household for attaining the objectives of the study.

As shown in Table 1, at the first stage two districts, i.e., Lahore and Faisalabad, were selected from the province of Punjab. At the second stage, two towns were selected from both districts randomly. At the third stage, four union councils (UCs) and three municipality wards were selected randomly from each town. At the fourth stage, ten students were randomly selected from each union council and municipality ward, respectively. Total sample size, thus, became 240 households. In this study, the questionnaire consists of the household information, working and not working members, higher secondary level college/school-going child information and whether he is taking private tuitions or not and how many subjects they are taking. These are as follows:

**TABLE 1:** Selection of sample size.

Districts	Lahore	Faisalabad
Towns	2 (Allama Iqbal + Samanabad)	2 (Lyallpur + Jinnah)
Union Council/Municipals Committees	6	6
Student Sample	10	10
Sub total	2×6×10 = 120	2×6×10 = 120
Grand Total	120 + 120 = 240	

Source: Own illustration.

##### Household information

In this portion, we discussed the socioeconomic status of households. It includes the information about household size, male in household, female in household, working members of a household, working male and working female in house, and family system.

##### Working members

In this section, information about the working members in a household states about their economic status. In this table, we comprised details about household gender (male, female), age, qualification, occupation, and their estimated income they earned in a month. From this table, information regarding family income is taken.

##### Not college/school-going and not working

A house contains a family, and family contains multiaged members. It may include children below 14 years or grandparents having age above 70 years who neither do a job nor go to higher secondary level college/school. In this table, detailed information about the family members who are not going to college/school at the higher secondary level, not working, their gender, age, qualification, and any kind of allowances is given.

##### Higher secondary college/school-going

In this study, our research is mainly done on first higher secondary college/school-going children. The expenditures spent on a college/school-going child by the parents or working members of a household are the part of this table. It largely covers the information about the gender, age, class/grade, college/school type, cost of transport, and cost of stationary.

##### Do you (your child) take tuition

For children who are taking tuitions, information is asked related to the tutor, the amount of expenditures spent on shadow education, and the number of subjects being taught in private tutoring. In this table, information includes the name of subjects taken by child for tuition, type of tutor, the reason of taking shadow education, time spent on tuition, number of days in a week for tuition, duration of tuition in a year (whether the student takes tuition during summer vacations or not), number of students with same tutor, tuition fee, stationary cost in tuition, cost of transport, religious education, and time spent on religious education.

##### If not taking tuition

Some students do not take tuition during study. They use different means of study to fulfill their need of tuition. In this table, certain variables include which describe their means of study such as while studying at home their time of study, means of study (self or any other household), and their reason for not taking private tutoring.

##### Outcome/result

When there is input, there must be an output. If parents are spending their earnings as input, then they must expect best grades as output, but it also creates economic burden of a family. Children taking private tutoring are supposed to show good rather than the best results in their studies. Lastly, a table consists of information about the results and economic burden of a family of the students.

#### Variables and their descriptions

The dependent and independent variables that are used in the study are the following:

##### Shadow education

In first, Hurder model dependent variable is used which is shadow education (student takes tuition or not), whether higher secondary college/school students taking tuitions or not (1 represents taking tuition, 0 for not taking tuition).

##### Economic burden

In the second, linear regression model dependent variable is economic burden. It is equal to tuition expenditure over family income. Student who takes tuition means they are the reason for economic burden of a family.

##### Region

Region means whether the household lives in rural or urban areas. Region is used as an explanatory variable to check its impact on demand for shadow education. This is measured through 1 for MC and 0 for UC. MC shows urban area, while UC indicates rural areas.

##### Gender

In gender variable, male and female category is used as 1 for male and 0 for female. The gender variable is also expected to affect economic burden of a family and demand for shadow education.

##### Family size

This explanatory variable consists of total members in a house that are grandmother, grandfather, mother, father, aunts, uncles, and children. It also affects the economic burden of a family and the demand for shadow education.

##### Parent’s education

Parent’s education consists of father and mother education. Parent’s education is taken as explanatory variables presenting the awareness regarding education. In this study, the level of education for parents is taken below matric and matric and higher education. This is the same as for mother education. Parent’s education effects economic share; the reason is that the syllabus of higher secondary classes is becoming tough and launched in English medium. So, parents having qualifications below matric and up to matric cannot teach their children well at the higher secondary level.

##### Family system

It defines whether household is living under the joint family system or separate. It is also an independent variable (one for joint family system and zero otherwise). It also affects the economic burden of a family and the demand for shadow education.

##### Tuition expenditures

Tuition expenditure on the first higher secondary school-going child is the independent variable. It represents the household demand for shadow education for their college/school-going child. The amount of money spent by parents on shadow education becomes tuition expenditures. These expenditures contain tuition fee, expenditure on stationary, and transport expenditures. These expenditures show the household willingness to pay for shadow education.

##### College/school type

This is also an independent variable showing the types of college/school as government and private schools (1 for private and 0 for government).

##### Working member

This explanatory variable consists of total working members in a house that are mother, father, aunts, uncles, sister, and brother. Working members in a family share the economic burden of a family.

##### Father occupation

This explanatory variable consists of father occupation. In this explanatory variable, 1 indicates government job, 2 indicates private job, and 3 indicates own business. Fathers’ occupation also affects economic burden of a family and the demand for shadow education as father’s job is the major source of income in every family.

## Results and discussions

This section gives descriptive statistics analysis of the collected data that contains socioeconomic status of household, education level of household, private tuitions taken by students, students age, grade, and gender, patterns of tutoring, and type of tutor or providers. Section “Research methodology” provides analysis of Hurder model for analysis of regional factors which means family, individual, and school factors and their econometric interpretations. Section “Results and discussions” provides an analysis of linear regression model for economic burden of people in the Lahore and Faisalabad districts of Punjab province.

Table 2 illustrates socioeconomic status of sampled household. The economic status of a family is directly influenced by household size. In most parts of Asia, Family size is increasing, and shadow education is inversely correlated with family size (Kim et al., 2022). A child with fewer siblings received more tutoring than of children with more siblings. In Table 2, column 1 shows household member of a family. Column 2 shows household size of a family, and column 3 shows school/college-going children of a family. The study shows that 24.2% of household size consists of 4 members and the percentage of school/college children is 54.3%. Further, the study shows that the number of school/college-going students is more in families where household members are less. The study illustrates that 58.9% of household size consist of nine members as Pakistan reached at the fifth number at the list of most populated countries in the world. We see that school/college-going children percentage is 41.5% in the big families, and it is less when compared with the small families as above. The study also reveals that 16.9% of household size consist more than nine members and the student of these families going to school/college is only 4.2% which is more less as compared to small families. The study reveals that the percentage of household size is greater than all, and the percentage of school/college-going is less than all. As per this table, it is concluded that more members of a house have less capacity to spend on the education of their children.

**TABLE 2:** Socio economic status of household.

House hold member	House hold size	School/College going children
1–4 member	24.2%	54.3%
5–9 member	58.9%	41.5%
More than 9	16.9%	4.2%

Source: Own illustration.

Table 3 illustrates the education level of household. The findings of the study suggest that private tutoring is not accessible to all students (Silova, 2009). After analyzing the data, it is evaluated that the education of household influences the private tutoring of children. In this table, we explain the education level of household with their students. After evaluation, we see that father education in Punjab province is more than mother education in household. Traditionally, females (mothers) take less education in Punjab than males (fathers). The Annual Status of Education Report [ASER] (2016) also has shown that in Punjab mothers are more illiterate than that of fathers. In Table 2, father education below matric is 14.3% and mother education is 32.6%. The study shows that mother is more illiterate than father. Mothers having education below than graduation are 45.3%, and fathers are at 29.2% in this section. According to this study, it seems that father education is higher than mother education. The study evaluates that in higher education mother education is 22.1% and father education is 56.5%. According to formal socialist bloc, it is likely that better education parents recognized the potential benefit of investment in private tutoring and other courses.

**TABLE 3:** Education level of household.

Education intensity	Father education (%)	Mother education (%)
Below matric	14.3%	32.6%
Below graduation	29.2%	45.3%
Higher education	56.5%	22.1%

Source: Own illustration.

In this table, the study finds that most of the students take private tuition. Limited students are not taking tuition for their studies. There are 57.1 percent of students taking tuition from total sample size 240, while 42.9 percent of students are not taking tuition. The study finds the importance of shadow education in the mainstream system. The location has a great impact on shadow education. At the society level, urban students commonly receive more shadow education than their rural counterparts. Underlying reasons are linked to both demand and supply (Tansel and Bircan, 2006; Dang, 2007; Kim, 2010; Bray, 2011). Such society which lives in urban areas takes more tuition than those living in rural areas. In urban areas, there are more opportunities to get private tutoring than in rural areas. Likewise, there are more tuition centers with easy access than in rural areas. Parents are more educated in urban areas, and awareness is playing the key role in product of education. Urban life is very competitive than rural life. In socioeconomic term, urban parents are better to afford tuition for their children. Similar remarks have been made with reference to Greece (Polydorides, 1986) and Egypt (Fergany, 1994).

We see in this table there are 32.6 percent taking tuition from urban areas and 24.5 percent taking tuition from rural areas. In the given table, difference of (8.1 percent) exists between the demand for shadow education in both rural and urban areas. In the same way, Lahore district (Allama Iqbal Town and Samanabad Town)-wise analysis shows that in Lahore 33.4 percent of students are receiving private tutoring while 17.3 percent of students are not taking tuitions. Similarly in Faisalabad district (two towns, i.e., Lyallpur and Jinnah), 23.7 percent of students are receiving private tutoring and 25.6 percent of students are not taking tuitions.

In Table 4, descriptive analysis of school/college level shows that 66.4 percent students of private institutes are getting benefits of private supplementary tutoring while 33.6 percent of students in government schools. There exists a great difference of 32.8 percent between government and private schools for the demand of shadow education. It showed that the incidence of taking shadow education is larger in private schools as compared to government schools. It is just because parents are unable to cope with the tough syllabus of private schools. Our analysis of the data matches with the findings of Annual Status of Education Report [ASER] (2016) survey of Punjab which indicated that the demand for private tuition remains higher in private school students as compared to their counterparts.

**TABLE 4:** Private tuition by students as Society level and School/College Type.

Sample size *n* = 240	Overall percentage	Tuition-taker (%age)	No tuition-taker (%age)
Society level	100%	57.1%	42.9%
Urban	50%	32.6%	14.5
Rural	50%	24.5%	28.4
Lahore district	50%	33.4%	17.3%
Faisalabad district	50%	23.7%	25.6%
**School/College type**			
Government	40%	33.6%	61.7%
Private	60%	66.4%	38.3%

Source: Own illustrate.

Generally in the Lahore district, there are more private school/colleges than government schools/colleges. In Faisalabad district, there is less private school/college than Lahore district. A survey occurred in 2010 found that 25.3% of students aged 6–16 in private schools received private supplementary tutoring compared with 9.7% in government schools (Annual Status of Education Report [ASER], 2011).

The study also finds that 61.7 percent of students are not taking tuition of government school/college and 38.3 percent of students are not taking tuition of private school/college. There is also a gap between government and private school/college which is 23.4 percent. Most studies on Pakistan find a positive and significant learning gap between private and public schools (Khan and Shaikh, 2013).

The analysis of Table 5 shows the gender inequalities. In this table, descriptive analysis of participation of shadow education is clearly visible. In Table 5, the statistical data show and designate the sociocultural features of the gender gap between girls’ and boys’ contribution in private tutoring. Participation in shadow education may vary by gender. In some countries, parents tend to spend more on boys’ tutoring (see, e.g., Sujatha and Rani, 2011; Tansel, 2013). When parents have to make decisions on whether to invest in the tutoring of boys or girls, they are more likely to choose the former on the grounds that boys because they are more likely to seek paid employment that will require educational qualifications. Gender inequalities have been found at secondary levels.

**TABLE 5:** Private tutoring incidences by gender, grade level, and subject wise.

Total sample size *n* = 240	Tuition taker percentage	Not tuition taker percentage
**Gender**		
Male	55.4%	38.7%
Female	44.6%	61.3%
**Grade**		
11th grade	53.7%	46.3%
12th grade	46.3%	53.7%
**Subjects**		
English	10.4 %	89.6%
Math	20.0%	80%
Physics	46.7%	53.3%
Chemistry	27.5%	72.5%

Source: Own illustration.

As guys are too expected to run their family, so a brilliant scholastic record is viewed as the assurance of splendid future. Subsequently, a particular consideration is paid to the instructive necessities of young men. Therefore, specific attention is paid to the educational needs of boys. The given table shows that 55.4 percent of male students participated in tutoring, while 44.6 percent of female students received it. It presents a substantial variation (10.8) between the amount of male and female participation during the last year.

In this table at grade level, we take only data of secondary school students which means we take only 11th and 12th grade of students. Our research found that at higher secondary level, private tutoring is found more than other levels. Aslam and Atherton (2012) also found that there is huge intensity of private tutoring, especially in Lahore. In the above table, we see that 11th-grade students take more private tutoring than 12th grade. We see those 53.7 percent students of 11th grade and 46.3 percent student of 12th grade take private tutoring because the students of 11th were grade more energized after matriculation and they tried their best at every cost. That is why, from the very first day, they attend their college classes; additionally, private tutoring plays its role in compensating for a deficiency in their studies.

We see in Table 5 the demand for private supplementary tutoring are those subjects that are most necessary for advancement in the education systems of Punjab province, especially in Lahore and Faisalabad districts. This usually includes English, math, physics, chemistry, and biology. It is also observed that students take more private tutoring of science and math subjects at the higher secondary level (Silova, 2009). The study finds that 46.7 percent of students take private tuition for physics subjects, and it is really high in Lahore district. It is also found that 27.5 percent of students take tuition for chemistry subject. It is at 2nd number in our study. We also see that 25.0 percent take tuition for biology subjects. A survey collected in Pakistan in 2011 found that there is huge intensity of private tutoring in science subjects, especially in Lahore and Faisalabad.

We also see that 20.0 percent of students take mathematics subject tuition and 10.4 percent of students take English subject tuition. Zhang’s survey in Jinan, China, showed lower rates of students receiving tutoring in mathematics. In our study, we find that students take more science subjects tuition than others. In the shadow education system, English as a subject has received little attention in the research literature (Bray and Kwok, 2003, p. 614). According to our analysis, English is the subject with the strongest demand for supplementary tutoring since. It is not only a key academic subject, but it is also used as medium of instruction for other subjects in many schools. Parveen and Phuc (2020) also identified similar reasons that forced students to join private tuition in Pakistan as to gain help for removing the difficulties they face in particular subject (e.g., mathematics, physics, chemistry, etc.).

There are different patterns of private tutoring in the world. Pattern of private tutoring is not universal (Aslam and Atherton, 2012). The survey in the Jinan, China, by Zhang (2013) found that mostly students take private tutoring in one-to-one and small groups.

In Table 6, there are three types of private tutoring that are documented among the patterns of private tutoring. At one end of the scale is individual tutoring, one-to-one tutoring, commonly delivered in the homes of either the tutors or their students. Alternatively, pupils may receive tutoring in small, medium-sized, or large groups. A small-group tutoring that includes two to nine students is the most common among the students. Maximum proportion, 49.3 percent, of students in both areas selects small-group tutoring. Parents chose small-group tutoring because they wanted more attention from tutors for their children or chose tutors in their own village or city streets due to security threats. The second most popular tutoring mode is class-style or large-group tutoring which contains more than 9 students, and 42.8 percent of students are obtaining this type of tutoring. One-to-one form of private tutoring has the lowest percentage, 3.05 percent, of students. This type of tutoring is expensive for many parents that is why less demand is from them.

**TABLE 6:** Patterns of tutoring.

Patterns of tutoring	Percentage (%)
Individual tutoring (one-to-one)	7.9%
Small-group tutoring (2–9 students)	49.3%
Large-group/class style tutoring (10+students)	42.8%

Source: Own illustrate.

### Results of empirical analysis

Table 7 represents the Hurder empirical analysis of demand for private supplementary tutoring in which an explanation of the factors affecting the demand for private supplementary tutoring is presented. This empirical analysis contains and describes the factors effecting household decisions to take private supplementary education for first higher secondary/college-going children.

**TABLE 7:** Hurder model.

Independent variables	Co-efficient B	Standard errors SE	Significance *P*-value
Rural UC/Urban MC = 1	2.335*	2.374	0.025
House hold size	1.288**	0.697	0.064
College going	–2.626*	1.729	0.023
Family System1 = joint	–3.183***	1.937	0.100
Family income	1.480*	2.678	0.009
Gender1 = Male	0.577**	1.153	0.062
College type1 = Private	814***	1.239	0.093
Father education	Reference Category		
Father education (No education)	–1.679***	1.854	0.567
Father education (Up to Matric)	2.343***	1.598	0.163
Father education (Higher education)	–3.345**	2.008	0.078
Mother education	Reference Category		
Mother education (No education)	1.563***	2.322	0.432
Mother education (Up to Matric)	–2.34**	1.788	0.094
Mother education (Higher education)	–3.527*	2.101	0.032
Father occupation	Reference Category		
Father occupation 1	4.302***	1.159	0.801
Father occupation 2	1.015**	2.041	0.64
Father occupation 3	3.042*	1.04	0.21
Constant	–3.152	1.852	0.597
*N* = 240, Pseudo *R*^2^ = 0.432.			

*P < 0.5, **P < 0.10, ***P < 1.00.

#### The demand for private tutoring: Determinants of probability in different contexts

In the Hurder model, determinants of probability of receiving private tutoring are presented. This analysis contains regional analysis, family analysis; individual analysis, and school analysis of these factors (Table 7).

##### Regional factors

Regional- or community-level analysis illustrates that region/area has a positive but significant impact on the demand for shadow education. In Pakistan, a study of grade 12 pupils in a national sample found that the student of urban areas received private supplementary tutoring more than rural area students (Aslam and Atherton, 2012). When people move from rural to urban, the probability of taking shadow education for higher secondary classes increases by 2.335 units. Significant impact means that the chances of receiving private tuitions are highly affected by regional analysis. In urban areas, students take tuition due to several reasons; one is higher level of competitiveness among urban students. In urban areas, parents are also educated and have higher educational attainment, logical extension, and higher achievement expectations. Similar remarks have been made with reference to Greece (Polydorides, 1986) and Egypt (Fergany, 1994).

##### Family factor

This analysis consists of household size, number of college-going children, family income, family system mother education, father education, father occupation, and mother occupation. Household size, number of college-going, and family system have significant impact on demand for shadow education. In other words, the decision to take private tutoring is determined by regional and some family factors. Mother education at higher education level has positive impact on shadow education, but below higher education level in which up to matric education involved has insignificant impact on shadow education. Father education at higher education has significant impact on shadow education, but no education of father and up to matric education has insignificant impact on shadow education. Father’s occupation also has an insignificant impact on shadow education except if the father has his own business.

In the above-given table, we see that with the increase in household size demand for shadow education increases by 0.064 unit. It may illustrate that even large families are demanding shadow education for their children because some sort of tutoring is affordable for them. This result also explores that households try to secure this broadly treated necessity anyway (Bray and Kwo, 2013). College-going has significant influence on the probability of taking private tutoring. This analysis shows that as the number of college-going children increases the chance for receiving private supplementary tutoring is reduced by 0.023 units. In this analysis, it shows the inverse relationship between college-going and shadow education. Research in Taiwan, Vietnam, and Philippines also proved the inverse relationship between the demand for private tutoring and number of children in a family (Dang and Rogers, 2008; Liu, 2012; Fakih et al., 2022).

Family income has significant and positive associations with the probability of taking private supplementary tutoring. When family income increases, the probability of taking private tutoring also increases one time more than the others having less income. Mostly analysis shows positive correlation between family income, and private tutoring was consistent with the empirical studies cited above (Tansel and Bircan, 2006; Ho et al., 2008; Chu, 2009; Kenayathulla, 2012; Jang, 2018; Cheng, 2022). Children in higher socioeconomic groups generally receive more supplementary tutoring than children in low-income group (Stevenson and Baker, 1992; Nath, 2008). Family system has significant impact on shadow education. Family system as a joint has inverse relation with shadow education. As the number of family members increases the chance for private tutoring and supplementary tutoring is reduced by 0.100 units. When family member increases, the household size of this family increases and, on the other hand, negatively affects the demand for private tuition (Tansel and Bircan, 2006).

We interpreted coefficient and estimates variables regarding the level of father’s education that a father having no education or primary-level education, below than matric, up to matric education, higher education, and inter- to graduate-level education involved. In this analysis, we estimate that father having no education has insignificant and negative relation with shadow education. The analysis shows that as father have no education it will reduce to get private tuition by 1.679. According to the economic survey 2015–2016 of Pakistan, there are an estimated 22 million (25+ years) illiterates in Pakistan. Father’s education has an insignificant impact on students’ private tuitions. The possible reason may be that educated fathers are mostly job holders, which means they are not available for their children to give them tuitions. In analysis, we see that as increase in father education there are 3.345 units reduced in getting private tuition. Similarly, we also interpreted coefficient and estimates explanatory variable regarding mother education. In this regard, if mother has no education or only literate, it is insignificant and has positive correlation with shadow education. They do not give time to their kid’s education at home due to less education; therefore, there is a 1.563 demand for shadow education. Another variable is up to matric education, and it has a significant and inverse relation with shadow education. Here, we see that at the primary-level mothers teach their kids but up to matric they do not teach their children because of tough syllabus. So there is also 2.34 unit demand for shadow education. We see in our analysis that higher education of mother has a significant impact on shadow education. Mothers having higher education are busy in jobs; therefore, they do not teach children, and there is 3.527 unit demand for shadow education at the higher secondary level. Studies suggest that households with an educated mother are more likely to value education and place greater importance on children completing their schooling, perhaps, because a literate woman is more empowered and has higher bargaining power at home. Therefore, the mother’s level of education can be used as a proxy for the household environment (Kenayathulla, 2012). Accordingly, there seems a higher probability that the students with more highly educated parents will take shadow education. The result is entirely consistent with earlier research by Xue and Ding (2009); Kim and Lee (2010), and Zhang (2013).

Father occupation has less significant impact on shadow education. We also interpreted that father occupation as government job, private job, and own business. In our analysis, we see that government job holder father’s children take tuition less than private job holder father and all are less than those who have their own business. Our study finds that own business father has more income than private job and government job; therefore, the children of own business father demands more private tutoring. It has significant and positive relation with shadow education. Here, demand for shadow education is 3.042 which is more than other. Government occupation father also demands for shadow education but less, and it is insignificant. Private occupation is also significant and 1.015 unit demand for shadow education of their children. Occupation has a significant impact in the context of income.

##### Individual/student factors analysis

Individual or student analysis contains gender-level factors. Our analysis results show that the probability of private tuitions for male is 0.577 units more than for female, which demonstrates that gender inequality exists in the data due to male-dominated society. Boys are preferred while taking the decision for shadow education as compared to their counterparts. This result is consistent with the earlier studies that are showing gender disparities in their sampled data (Nath, 2008; Sujatha and Rani, 2011; Aslam and Atherton, 2012; Tansel, 2013). Parents tend to spend more on boys’ tutoring (Sujatha and Rani, 2011; Tansel, 2013).

##### College/school factor analysis

College/school-type has significant impact on making decision to get private tutoring. The probability of private tuition of students of private college or school is 0.814 units more than government school students. Our analysis describes that private school students take more tuitions as compared to government schools. School-type is a stronger predictor of demand for shadow education (Khan and Shaikh, 2013; Zhang and Bray, 2015; Bray, 2020).

The students in private schools consume more on private tuition than government school students. It is due to the fact that as compared to government schools structure private school system is well organized and well established. Less attention is paid by government school teachers in mainstreams due to more strength and less availability of teachers (Kim, 2004; Zhang, 2013).

### Linear regression model

Section “Results and discussions” describes the third model, an analysis of linear regression model for economic burden of people in Punjab province. Table 8 represents the linear regression model analysis of economic burden for shadow education for the first college-going child. This analysis describes the factors which create economic burden for private tutoring. This is presented as under:

**TABLE 8:** Linear regression model.

Model	Un standardized coefficients		Standardized coefficients	*T*-values	Significance
	*B*	Standard error	Beta		
(Constant)	2.653*	1.753	1.689	1.513	0.0132
UC/MC 1 = Rural	0.082**	0.163	0.021	0.501	0.073
Household size	0.082**	0.059	0.080	1.402	0.062
Gender 1 = Male	–3.034	2.001	0.989	3.656	0.098
College going	0.478***	0.195	0.143	2.457	0.015
Working member	0.109***	0.092	0.054	1.184	0.38
Age	–0.262	0.127	–0.135	–2.064	0.040
Grade	0.293***	0.235	0.084	1.246	0.214
College type 1 = private	0.300***	0.161	–0.081	1.860	0.064
Other expenditure	0.301***	0.240	0.280	2.085	0.923
Mother education	–1.042	0.339	0.654	–3.762	0.76

*P < 0.5, **P < 0.10, ***P < 1.00. Number of observations = 240. *R*^2^ = 0.2340.

#### Regional factors

In this model, urban and rural are regional factors. In Table 7, urban and rural have significant impact on economic burden. The quality of education in rural schools and colleges may not be too satisfactory. Students must get private tutoring or any mentor to compensate for their studies. It may lead to creating an economic burden on the family. The rural factor is significant (0.82 units) have a high possibility of creating an economic burden. Private tuition also plays an important role in declining the quality of education provided in government institutions. It is observed that the private tuition has a negative effect on regular schools, including negatively effecting teacher’s performance, their way with students and families (Bray, 2013). Some of the negative effects of private tuition on individual students and their families are the economic burden on family income and the social necessity or trend of joining private tuition even when it is not necessary for some students (Fakih et al., 2022).

#### Family factor

This analysis consists of household size, number of college-going children, working member of a family, and mother education.

This analysis shows that household size very strongly affected and impacted on economic burden. As the number of household members increases, it creates economic burden and also impacts on students’ private tuition and their families (Hajar, 2018; Jang, 2018). It results in positive and effects negatively on the social and psychological lives of Pakistani people, especially students and their families (Kenayathulla, 2012; Khan and Shaikh, 2013). We see that as household member of a family increases there is increase in economic burden 0.062 unit (in terms of expenditure). We see that while considering household size data find the positive and significant correlation between household size and economic burden in terms of private tutoring. The study illustrates that even large families are demanding shadow education for their children, and it creates economic burden. This result also explores that households try to secure this broadly treated necessity anyway (Bray et al., 2014). The work of Tansel and Bircan (2006) in Turkey also supported the fact that as the number of children boosts the expenditure, shadow education is decreased by parents.

Further, college-going has significant influence on the economic burden. This analysis shows that as the number of college-going children increases economic burden of that family also increases by 0.478 units in terms of expenditures. It is also related to the literature (Dang and Rogers, 2008; Liu, 2012). Likewise, working members of a family have significant impact on the economic burden. When working member of family increases, income also increases; therefore, the probability of economic burden of the family decreases with 1.109 units. Working members are correlated with socioeconomic groups and generally receive more supplementary tutoring than the children in low-income group receive (Stevenson and Baker, 1992; Dang, 2007). It means that economic burden is also related with high-income group and low-income group. The students belonging to all socioeconomic classes (low-, middle-, and higher-income groups) and all educational levels (from primary schools to the university level) join private tuitions in Pakistan (Parveen and Phuc, 2020). Our analysis shows those high-income groups have more working members, and economic burden of these families is less and vice versa.

Mother education has significant impact on economic burden. Our analysis shows that highly educated mothers go to job but do not teach their children; therefore, economic burden still remains as well because they do not teach their children. They prefer to do jobs and pay tuition fees; in this way, it creates economic burden. Mother education decreases in economic burden with 1.042 only when mothers are educated and they teach their children and do not pay tuition fees. Some parents are uneducated or illiterate, and secondly, some parents are educated but have no time and remain too much busy to improve their economic position (Parveen and Phuc, 2020).

#### Individual/student factors analysis

Individual or student analysis contains gender-level and other expenditures. Gender inequalities exist everywhere and are found in educational matters also. Gender analysis shows that female demands more for shadow education as compared to male; in Pakistan, it assumes an economic burden. Expenditure on private tutoring for male is 0.098 units less than the female. This result matches with Zhang’s study (2013) in Jinan, China, describing that there is larger proportions of females receiving private tutoring. In Pakistan, male students take more tuitions than female. This result is consistent with the earlier studies that are showing gender disparities in their sampled data (Nath, 2008; Sujatha and Rani, 2011; Aslam and Atherton, 2012; Tansel, 2013). It is also disclosed that parents tend to spend more on boys’ tutoring (Sujatha and Rani, 2011; Tansel, 2013).

Other expenditures have a great and significant impact on shadow education and economic burden; we see that as expenditures of students increase, there is also increase in the economic burden of the family. Other expenditures include transport cost and stationary cost in face-to-face tutoring and internet cost for online tutoring. When expenditures increase, the economic burden of a family also increases by 0.923 units. It is significant and also matches with previous literature (Bray, 2013) (Zhang and Bray, 2015; Zheng et al., 2020).

#### College/school factor analysis

College/school factor also has a significant impact on the economic burden of a family. Students who study at private colleges are the major cause of economic burden and vice versa. The students in private schools consume more on private tuition than government school students. It is due to the fact that as compared to government schools structure, private school system is well organized and well established. Less attention is paid by government school teachers in mainstreams due to more strength and less availability of teachers (Kim, 2004). Students’ class-level plays a vital role in student’s life, but it has insignificant and positive relation with economic burden; we may say that with the rise of class level, economic burden rises too on a family. But it is insignificant in this study (Kim and Lee, 2010).

Individual student is one who has eye contact with a private tutor and goes to tuition centers, directly. It is found that the private tuition centers have social and psychological impacts on students and are reason for economic burden. The data analysis shows that private tuition has social and psychological impact on individuals, their families, and the broader society in Pakistan (Kim, 2010; Crawford, 2011; Liu, 2012; Jang, 2018). It is observed that a substantial portion of family/household income is being spent on the cost of acquiring private tutoring in different countries (Silova, 2009). Hajar (2018) argued that it causes a considerable financial burden on parents.

## Conclusion and recommendations

Although there is extensive research regarding economic burden of private tutoring (Kim et al., 2022), demand for private supplementary tutoring, contributing factors, and their effects are investigated in a few studies so far (Benckwitz et al., 2022). The present study therefore adds to the body of research the demand for private tutoring, its contributing factors, and their effects in the context of Punjab province of Pakistan. Further research is needed to test whether these findings can be generalized across countries that have differences in the extent to which private tutoring takes place in their country and they also differ in their education systems (Tansel, 2013).

Shadow education has long been visible in East Asia; in recent years, it has greatly expanded and has evolved in format. In this study, we found that there is high price discrimination in tuition expenditures. On the other hand, almost 57.1 percent of the respondents take shadow education. The demand is higher for private school students rather than government students. It means that demand for shadow education is increasing, but there is no price regulation. So, there is a need to regulate markets for shadow education which could set the equitable price system for each consumer. In the short run, shadow education has significant impact on the students’ academic outcomes, and sometimes, it is used for remedial measure. This result is in consistent with the previous studies given by Lauer et al. (2006), Crawford (2011), and Jang (2018). However, in the long run it may put burden on the household budget share which says that economic burden. Thus, policy-makers should set such educational policies that increase teachers’ and school performance in the long run. Therefore, the government should regulate the well-established academic system, and it ensures that at least the educators in both institutions, public and private, should be graduated with specific teacher trainings. So, they can teach their students efficiently in such a manner that each student would not need any supplemental education.

Similarly, level of education for parents (below matric) who has been set in this study found that literate mothers demand for shadow education for their children while fathers’ education has no impact on the demand for shadow education. It is shown that educated mothers spend more on the private tuitions. It might be that educated mothers work and have less time for their children so the demand for shadow education is increased. But mothers should have to teach their children at home regardless of sending them on tuitions. Likewise, large families are cause to economic burden of a family, especially the families having low income. Moreover, joint families have significant impact on demand for shadow education. Punjab Government should make such policies to provide financial support or any type of scholarships as kinship to large families’ students. Socioeconomic profile of household has been discussed which includes household size, family system, working members both male and female, and number of college/school-going children. Parents’ level of education and family income are also discussed as a significant determinant. Next, the type of tutoring from one-to-one to large classes and the type of tutor or supplier of private tutoring are also explored in the descriptive analysis. Hurder model results for demand for shadow education are examined to check which factors are affecting the demand for shadow education. Linear regression model is discussed for economic burden of a family whose children take tuition.

## Implications

Research findings will not only help parents to come down their expenses but also for the policy-makers for making regulate the prices of shadow education that can contribute to consumers for having benefits from shadow education.

## Data availability statement

The original contributions presented in this study are included in the article/supplementary material, further inquiries can be directed to the corresponding author.

## Author contributions

KP and ID presented the main idea and wrote the first draft of the manuscript. AA and IJ contributed to conduct the methodology. All authors proofread the manuscript before submission.
